# SARS-CoV-2 Delta Variant Pathogenesis and Host Response in Syrian Hamsters

**DOI:** 10.3390/v13091773

**Published:** 2021-09-05

**Authors:** Sreelekshmy Mohandas, Pragya Dhruv Yadav, Anita Shete, Dimpal Nyayanit, Gajanan Sapkal, Kavita Lole, Nivedita Gupta

**Affiliations:** 1 Indian Council of Medical Research-National Institute of Virology, Pune 411021, India; sreelekshmy88@gmail.com (S.M.); anitaaich2008@gmail.com (A.S.); dimpal.nyayanit@gmail.com (D.N.); gajanansapkalniv@gmail.com (G.S.); lolekavita37@yahoo.com (K.L.); 2 Indian Council of Medical Research, V. Ramalingaswami Bhawan, P.O. Box No. 4911, Ansari Nagar, New Delhi 110029, India; drguptanivedita@gmail.com

**Keywords:** SARS-CoV-2, Delta variant, B.1.617.2, B.1.617.3, Syrian hamster, pathogenicity

## Abstract

B.1.617 is becoming a dominant Severe Acute Respiratory Syndrome-Coronavirus-2 (SARS-CoV-2) lineage worldwide with many sublineages, of which B.1.617.2 is designated as a variant of concern. The pathogenicity of B.1.617.2 (Delta) and B.1.617.3 lineage of SARS-CoV-2 was evaluated and compared with that of B.1, an early virus isolate with D614G mutation in a Syrian hamster model. Viral load, antibody response, and lung disease were studied. There was no significant difference in the virus shedding pattern among these variants. High levels of SARS-CoV-2 sub genomic RNA were detected in the respiratory tract of hamsters infected with the Delta variant for 14 days, which warrants further transmission studies. The Delta variant induced lung disease of moderate severity in about 40% of infected animals, which supports the attributed disease severity of the variant. Cross neutralizing antibodies were detected in animals infected with B.1, Delta, and B.1.617.3 variant, but neutralizing capacity was significantly lower with B.1.351 (Beta variant).

## 1. Introduction

Severe Acute Respiratory Syndrome-Coronavirus-2 (SARS-CoV-2) B.1.617 lineage variants were first reported in India in October 2020. Among the reported sublineages, B.1.617.1 is designated as a variant of interest and B.1.617.2 as a variant of concern (VOC) by the World Health Organization [1]. B.1.617.3 is another sublineage in which fewer sequences have been reported. The rise in COVID-19 cases worldwide during the second wave was speculated to be due to the high transmission potential of the Delta variant, which replaced the other variants in circulation [2]. As of 10 August 2021, the Delta variant has been reported in 142 countries [1]. The characteristic mutations reported in the spike gene of the B.1.617 lineage are D111D, L452R, D614G, P618R, and ±E484Q [3]. These mutations suggest increased ACE2 binding, transmissibility, and escape of neutralization [3,4,5,6]. Available evidence suggests increased transmissibility, secondary attack rate, hospitalization risk, and immune escape by the Delta variant [1,7]. The potential impacts of the Delta variant on vaccine and therapeutic effectiveness are uncertain as limited data are available. Recent studies have reported neutralization efficiency in vaccinated individuals and resistance to monoclonal antibody therapy of the Delta variant [8,9,10,11,12].

Animal models have been used to explore various disease aspects of SARS-CoV-2 as well as for establishing the safety and efficacy of many interventional measures [13]. Studies have shown the high binding affinity of SARS-CoV-2 spike protein to hamster ACE2 receptor [14]. The virus replicates to high titre in the respiratory tract of Syrian hamsters and causes pneumonia [15]. The model has been utilized for studying SARS-CoV-2 pathology, immune response, and transmission, as well as many preclinical drug and vaccine trials [15,16,17]. With the emergence of new variants, it is important to generate information on disease characteristics and replicative fitness in the existing animal models. In the present study, pathogenicity and virus shedding differences of the Delta and B.1.617.3 variant in a Syrian hamster model were assessed. Cross-neutralization potential of sera of variant infected hamsters was also investigated. The study parameters were compared with that of B.1, an early SARS-CoV-2 variant.

## 2. Materials and Methods

### 2.1. Virus and Cells

SARS-CoV-2 variants Delta (GISAID identifier: EPI_ISL_2400521), B.1.617.3 (GISAID identifier: EPI_ISL_2497905), and B.1 (GISAID identifier: EPL_ISL_825084) were used for the animal study. For in vitro neutralization assay, B.1.351 (Beta variant) [GISAID identifier: EPI_ISL_2036294] was also used. The isolates were propagated and passaged twice in VeroCCL81 cells and sequence verified by next generation sequencing. The virus stock was titrated to determine the 50% tissue culture infective dose (TCID50)/mL.

### 2.2. Animal Experiments

To understand the virus shedding and pathogenicity, 54 female Syrian hamsters (8–10 weeks old) were divided into three groups of 18 animals each. The respective groups were challenged with 0.1 mL of 10^5^ TCID50/mL of B.1, Delta, and B.1.617.3 variants intranasally under isoflurane anesthesia. The animals were monitored for any clinical signs and body weight loss. Four hamsters were kept as mock control for the study. Throat swabs (TS), nasal wash (NW), and faecal samples were collected in 1 mL virus transport media (HiMedia, Mumbai, India) from six hamsters/group on 3, 5, 7, 10, 12, and 14 days post infection (DPI). To study the pathogenicity of each variant, four hamsters from each group were euthanized on 3, 5, 7, and 14 DPI by isoflurane anesthesia overdose to collect blood and organ samples.

### 2.3. Viral RNA Detection

Tissue samples were weighed and homogenized in 1 mL sterile media (GIBCO, Thermo Fisher Scientific, Waltham, MA, USA) and TS/NW/faeces samples were collected in 1 mL media. The same were used for RNA extraction using MagMAX™ Viral/Pathogen Nucleic Acid Isolation Kit as per the manufacturer’s instructions. Real-time qRT-PCR was performed for E gene of SARS-CoV-2 to determine the viral genomic RNA (gRNA) load [18]. To measure the amount of replicating RNA, sub genomic (sg) N gene RNA was quantitated using published primers [19]. 

### 2.4. Anti-SARS-CoV-2 Antibody Detection 

The serum samples collected on 3, 5, 7, and 14 DPI were tested for IgG antibodies by a hamster anti-SARS-CoV-2 IgG ELISA [20]. Plaque reduction neutralization test (PRNT) was performed to understand the neutralization ability of the sera of variant-infected hamsters with the Delta, B.1.617.3, B.1, and Beta variants in a biosafety level 3 facility [21]. Briefly, Vero CCL-81 (1.0 × 10^5^ cells /mL) was added per well in 24-well tissue culture plates. The cells were incubated for 24 h in a CO_2_ incubator to obtain a confluent monolayer. A fourfold serial dilution of heat-inactivated serum samples mixed with an equal volume of virus suspension containing 50–60 plaque forming units /0.1 mL was incubated at 37 °C for 1 h. The virus–serum mixtures were added onto the cell monolayers and incubated with intermittent shaking. The mixtures were aspirated from the wells after 1 h and an overlay medium was added. The plates were further incubated at 37 °C with 5% CO_2_ for 4 days. The overlay medium was decanted and plates were stained with 1% amido black for 1 h. Plaques were counted and PRNT50 was calculated using a log probit regression analysis by SPSS software (SPSS 15.0, SPSS Inc., Chicago, IL, USA).

### 2.5. Histopathological Examination

The lung samples collected during necropsy were immersion fixed with 10% neutral buffered formalin. The tissues were processed with routine histopathological techniques and stained by hematoxylin and eosin. The lesions were graded on a numerical scale from 0 to 4 as no abnormality (0), minimal (1), mild (2), moderate (3), and severe (4) based on its severity by blinded scoring. Lesions graded include vascular inflammatory changes like congestion, hemorrhages, perivascular and peribronchial mononuclear cellular infiltration, bronchial pathological changes, alveolar changes like consolidation, hyaline changes, oedema, pneumocyte hyperplasia and septal thickening.

### 2.6. Statistical Analysis

The data collected from the study were analyzed using Graph pad Prism software (GraphPad Prism version 8.4.3, San Diego, CA, USA). For statistical analysis, non-parametric Mann Whitney test was used and *p*-values less than 0.05 were considered to be statistically significant.

## 3. Results

### 3.1. Clinical Observations and Virus Shedding 

**Figure 1:**
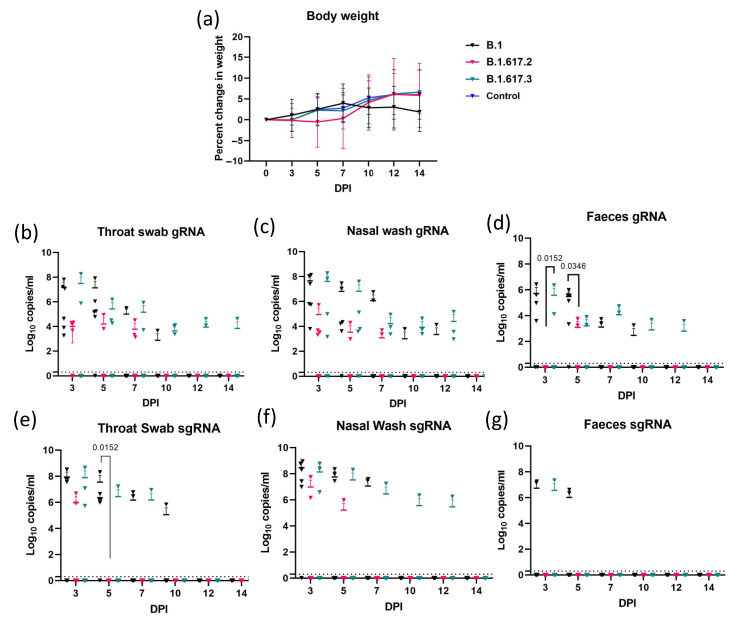
Bodyweight and virus shed by hamsters post challenge with SARS-CoV-2 variants. (**a**) Percent body weight changes in hamsters post virus infection at days 3 (*n* = 18); 5 (*n* = 14); 7 (*n* = 10); and 10, 12, and 14 (*n* = 6), as well as in mock control hamsters (*n* = 4). Viral genomic RNA load (log_10_ viral genomic RNA copy numbers/mL) in (**b**) throat swab, (**c**) nasal wash, and (**d**) faeces samples collected on 3, 5, 7, 10, 12, and 14 days post infection (DPI). Viral sub genomic mRNA load (log_10_ viral subgenomic RNA copy numbers/mL) in (**e**) throat swab, (**f**) nasal wash, and (**g**) faeces in hamsters post virus challenge on 3, 5, 7, 10, 12, and 14 DPI. The mean along with standard deviation is depicted in the scatter plot. The statistical significance was assessed using the non-parametric Mann–Whitney tests and *p*-values less than 0.05 were considered to be statistically significant. The dotted line indicates the limit of detection of the assay.

The average body weight gain in hamsters was the least in the Delta variant group compared with B.1 and B.1.617.3 during the first week of infection (Figure 1a). At 7 DPI, the mean percentage weight gain with standard deviation observed in the B.1, Delta, B.1.617.3, and mock control group was 6.72 ± 4.1, 0.3 ± 7.2, 2.15 ± 4.3, and 2.79 ± 3.15, respectively. For the Delta variant, the viral gRNA could be detected in the NW and TS samples until 7 DPI, whereas in a few animals of B.1 and B.1.617.3, it could be detected up to 12 and 14 DPI, respectively (Figure 1b–d). The viral load was higher in the TS and NW samples compared with faecal samples. The viral gRNA load was higher during the first week post infection, which decreased further in the B.1 and B.1.617.3 groups. After 5 DPI, sgRNA could be detected only in 1/6 animals in the B.1 group and 2/6 animals in the B.1.617.3 group and in none of the animals in the Delta variant group (Figure 1e–g).

### 3.2. Viral Load in the Respiratory Organs

**Figure 2:**
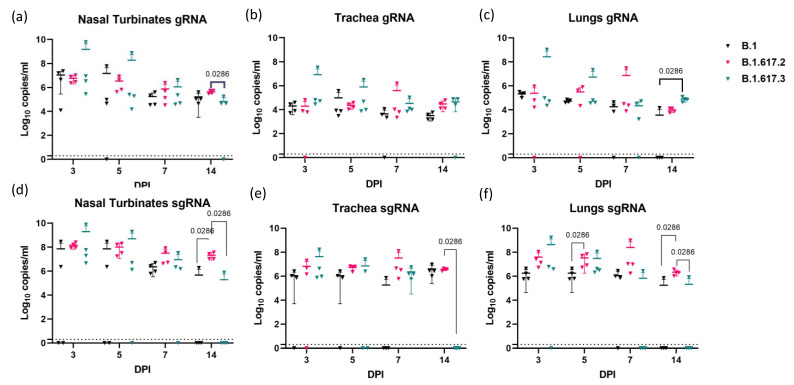
Viral load in respiratory tract samples of hamsters post infection. Viral genomic RNA load (log_10_ viral genomic RNA copies/mL) in (**a**) nasal turbinates, (**b**) trachea, and (**c**) lung samples collected on 3, 5, 7, and 14 DPI, represented as mean with standard deviation in a scatter plot. Viral sub genomic RNA load (log_10_ viral subgenomic RNA copies/mL) in (**d**) nasal turbinates, (**e**) trachea, and (**f**) lung samples collected on 3, 5, 7, and 14 DPI, represented as mean with standard deviation in a scatter plot. The statistical significance was assessed using the non-parametric Mann–Whitney tests, and *p*-values less than 0.05 were considered to be statistically significant. The dotted line indicates the limit of detection of the assay.

In the nasal turbinates, trachea, and lung samples, viral gRNA could be detected until day 14 (Figure 2a–c). Nasal turbinate showed a higher viral load compared with other organs in all of the groups. The SARS-CoV-2 viral RNA detected in the lungs, nasal turbinates, and trachea of hamsters infected with different variants did not show any statistical difference when compared among the groups on days 3, 5, and 7 post infection. By day 14, viral gRNA clearance was observed in 3/4 animals in the B.1 variant-infected group, whereas gRNA could be detected in all animals in the Delta and B.1.617.3 groups. The lung viral gRNA was significantly higher (*p* = 0.0286) in the samples of the B.1.617.3 variant on day 14 in comparison with B.1. The sgRNA could be detected in the lung and nasal turbinate samples until 7 DPI in all the groups. On 14 DPI, all the hamsters of the Delta variant-infected group showed significantly high sgRNA copies in nasal turbinates (mean = 2.1 × 10^7^/mL, *p* = 0.0286) and lungs (mean = 2.1 × 10^6^/mL, *p* = 0.0286), respectively, whereas in the B.1 and B.1.617.3 group, only 1/4 hamsters showed the presence of sgRNA (Figure 2d–f). 

### 3.3. Anti-SARS-CoV-2 Immune Response

**Figure 3:**
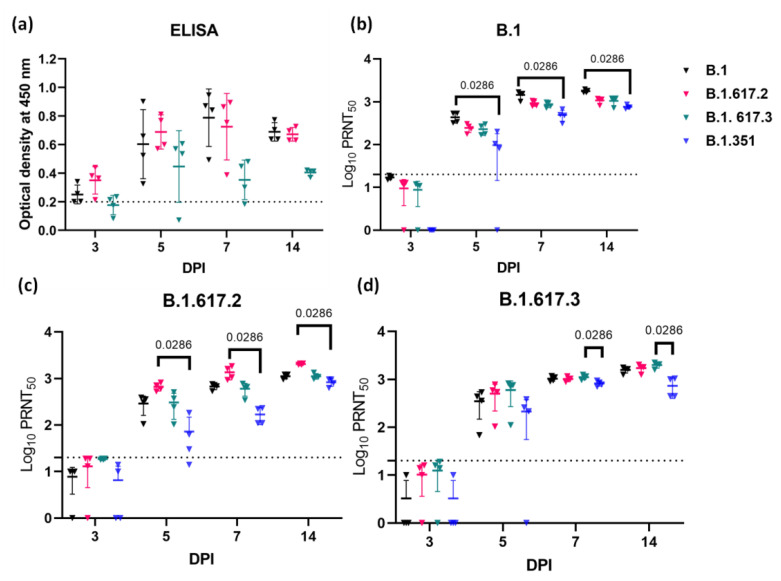
Antibody response in hamsters post challenge with SARS-CoV-2 variants. (**a**) Anti-SARS-CoV-2 IgG response in hamsters post virus infection by ELISA. Neutralizing antibody response in hamsters infected with the (**b**) B.1, (**c**) B.1.617.2 (Delta), and (**d**) B.1.617.3 variants against B.1, Delta, B.1.617.3, and B.1.351 (Beta). The dotted line indicates the limit of detection of the assay. The mean along with standard deviation is depicted in the scatter plot. The statistical significance was assessed using the non-parametric Mann–Whitney tests, and *p*-values less than 0.05 were considered to be statistically significant.

Anti-SARS-CoV-2 IgG antibodies were detected in all the groups from 3 DPI, showing an increasing optical density value in ELISA on further time points (Figure 3a). B.1 variant-infected hamsters showed about 1.7-fold geometric mean titre (GMT) reduction in neutralization titre against the Delta and B.1.617.3 variants, and 2.3-fold reduction against the Beta variant (*p* = 0.0286), on 14 DPI. In the case of Delta and B.1.617.3 variant infection, a significant reduction (*p* = 0.0286) in GMT against the Beta variant i.e., about 2.5- and 2.9-fold reduction, was also seen on 14 DPI. The GMT in the case of Delta variant-infected hamster sera was 1.8-fold reduced against the B.1 and B.1.617.3 variants, whereas the titre of B.1.617.3 infected sera was found to be 1.1-fold reduced against the Delta variant and 1.3-fold reduced against the B.1 variant on 14 DPI. 

### 3.4. Lung Pathology in Infected Hamsters

**Figure 4:**
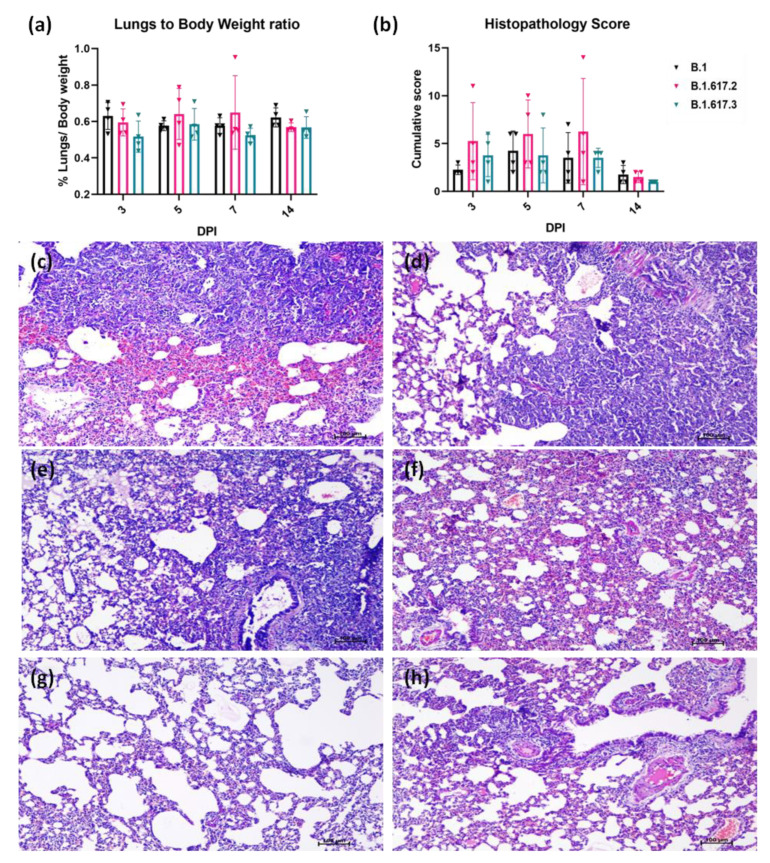
Pathological changes in lungs observed at necropsy in hamsters post infection with the B.1, Delta, and B.1.617.3 variants. (**a**) Proportion of lung weight to body weight of hamsters at necropsy represented as mean with standard deviation. (**b**) Cumulative lung histopathology score in hamsters infected with SARS-CoV-2 variants represented as mean with standard deviation. Lungs of Delta variant-infected hamsters showing (**c**) diffuse alveolar damage with congestion and hemorrhages in the lung parenchyma, as well as (**d**) diffuse mononuclear infiltration, pneumocyte hyperplasia and septal thickening with mild congestion. Lungs of B.1.617.3 infected hamsters showing (**e**) alveolar septal thickening, exudation and hyaline changes in the alveoli, as well as (**f**) congestion and alveolar septal thickening. Lungs of B.1 infected hamster showing (**g**) few small foci of mononuclear cell infiltration and (**h**) congestion and perivascular inflammatory cell infiltration.

Grossly, 2/16 of B.1, 6/16 of B.1.617.2, and 3/ 16 of B.1.617.3 infected hamsters showed congestion and hemorrhages. A slightly higher mean lung weight to body weight ratio was observed on day 5 and 7 in the B.1.617.2 infected hamsters (Figure 4a). The cumulative lung histopathological score also showed Delta variant-infected animals with a higher average score during the first week of infection (Figure 4b). Three/twelve of the Delta and 1/12 of the B.1.617.3 infected hamsters sacrificed during the first week of infection showed diffuse areas of consolidation, hemorrhages, pneumocyte hyperplasia, septal thickening, and perivascular and peribronchial inflammatory cell infiltration of moderate severity, and one hamster in the B.1.617.2 group showed severe lesions (Figure 4c**–**f). Lung tissues showed minimal to mild pathological changes in the case of all the B.1 variant-infected hamsters. The lesions observed were mostly focal on all days of sacrifice, except in 2/4 hamsters sacrificed on both day 5 and 7, which showed multifocal areas of consolidation and mononuclear cell infiltration (Figure 4g,h).

## 4. Discussion

The Delta variant possesses SARS-CoV-2 spike protein mutations that are known to affect virus transmissibility and neutralization. The secondary attack rate of the variant is also higher compared with the Alpha variant [7]. The variant is spreading faster across the globe to become a dominant variant in many countries [1]. We observed viral replication in the respiratory tract of hamsters post infection with the B.1.617 variant with minimal or no weight loss. Higher viral load in the throat and nasal swab samples was observed in the first week compared with faecal samples, indicating respiratory tract tropism. Earlier studies on various SARS-CoV-2 isolates of different lineages have also reported high viral loads in the initial week of infection in humans [22]. Viral sgRNA is considered to be an indicator of active infection [22]. We observed gRNA and sgRNA in the respiratory organs until 14 days. However, prolonged shedding of the virus could not be observed with any of the variants studied here, as reported in human cases [23]. The sensitivity of detection of viral sgRNA by nasal wash sampling post 1 week when viral gRNA load declines is less as per our observations in earlier studies in Syrian hamsters [16,24,25,26]. The prolonged detection of sgRNA in the nasal turbinates and lungs of B.1.617.2 could be a contributing factor to support the increased transmissibility attributed to this variant. Transmission experiments should be further performed to understand the significance of this finding. In human COVID-19 cases, where the presence of sgRNA is studied, it is found that it is rarely detectable post 8 days of illness [27,28]. A study from England has shown increased household transmission associated with the Delta variant compared with the Alpha variant, which was previously reported to be highly transmissible [29]. Increased transmissibility and immune escape have led to the sudden rise in COVID-19 cases due to the Delta variant [30].

Lung disease with severe lesions could be observed in the case of 4/8 Delta variant-infected hamsters sacrificed on day 5 and 7, indicating the potential of the variant to cause severe disease. The histopathological lesions observed were similar to the earlier reports in hamsters with an onset of inflammatory changes by 3 DPI, which progress to interstitial pneumonia and a complete recovery by 14 DPI [31]. Even though high levels of sgRNA and pneumonic changes could be detected in the lungs of B.1.617.2 infected hamsters compared with other variants, we could not observe any significant body weight loss post infection in hamsters as reported earlier, probably owing to the dose of inoculum. The minimal lesions observed with other variants could be also because of the same reason, as we have observed severe lesions and body weight loss in hamsters infected with a dose of 10^6.5^ TCID50/mL of the B.1 variant in our earlier studies [24]. The Delta variant has shown increased replication and enhanced entry efficiency in in vitro experiments [28]. An increased fitness advantage of the variant has been observed in the respiratory organoid system compared with wild type SARS-CoV-2 [28]. Reports from England and Scotland have shown an increased risk of hospitalization in the case of the B.1.617.2 variant cases [7]. Our earlier studies on pathogenicity of B.1.617.1, a variant of the same lineage in the hamster model, have shown severe lung disease [24].

The initiation of IgG response was observed as early as on 3 DPI. This finding was in contrast to our earlier studies in hamsters, where no IgG response could be observed on 3 DPI. This could probably be because of the difference in the virus inoculum dose and age of hamsters used [16,32]. The sample size of the study was also limited [16,32]. Day 3 sera were found to be non-neutralizing by the live virus neutralization assay. In neutralization studies, a significant reduction was observed in case of the Beta variant among all the variants studied. The Beta variant is known for reduced neutralization by many monoclonal antibodies and convalescent sera from patients infected with early SARS-CoV-2 isolates [30,33]. Although not significant, a reduction was also seen in the case of B.1 infected hamster sera with the Delta variant. Mlcochova et al., 2021 reported 20 to 55% immune evasion by the Delta variant in the case of prior infections with non-B.1.617.2 lineages [34]. B.1.617.3 possesses the E484Q mutation, which is a known site in RBD, which can impact the serum neutralization efficiency [6]. Even though we observed a reduced IgG antibody response in the case of B.1.617.3 compared with the other two variants at 14 DPI, we found the B.1.617.3 infected animal sera showing comparable neutralizing titres with the B.1 and Delta variants. Few recent studies reported reduced neutralization of the Delta variant by the BNT162b2 mRNA vaccine [8,9]. Another study reported only modest differences in the protective antibody titres against the Delta variant following two doses of the BNT162b2 or ChAdOx1 vaccine [10]. B.1.617 was found to be resistant to certain monoclonal antibodies approved for COVID-19 treatment such as Bamlanivimab and REGN10933 [11,12]. In India, sera neutralization studies on Covishield and Covaxin vaccine recipients showed neutralization potential against B.1.617.1 and B.1.617.2, respectively [32,35].

In the present study, we observed no significant difference in viral RNA shedding among the different variants studied. Higher sgRNA levels could be detected in the respiratory tract of Delta variant-infected hamsters for a prolonged period, which should be investigated further for its transmission potential. The Delta variant induced the least body weight gain and lung disease of severity in about 40% of infected animals, indicating its pathogenic potential. Cross neutralizing antibodies were observed in variant-infected hamsters. The neutralizing response was considerably lower with the B.1.351 variant. The evidence from the present study shows that Delta variant infection generates a broader neutralizing response and is not as immune-evasive as B.1.351.

## Data Availability

All the data pertaining to the study are available in the manuscript.
